# Evaluation of a virtual coaching system eHealth intervention: A mixed methods observational cohort study in the Netherlands

**DOI:** 10.1016/j.invent.2022.100501

**Published:** 2022-02-05

**Authors:** Marian Z.M. Hurmuz, Stephanie M. Jansen-Kosterink, Tessa Beinema, Katrien Fischer, Harm op den Akker, Hermie J. Hermens

**Affiliations:** a Roessingh Research and Development, eHealth department, Roessinghsbleekweg 33b, 7522, AH, Enschede, the Netherlands; b University of Twente, Biomedical Signal and Systems group, Drienerlolaan 5, 7522, NB, Enschede, the Netherlands; c VU University Amsterdam, Faculty of Human Movement Sciences, Van der Boechorststraat 7, 1081, BT, Amsterdam, the Netherlands

**Keywords:** Virtual coaching, eHealth, Older adults, Healthy lifestyle, User experience

## Abstract

**Background:**

With the rise in human life expectancy, the prevalence of chronic disease has increased significantly. Adopting a healthy lifestyle can decrease the risk of chronic disease. Virtual coaching systems can help older adults adopt a healthy lifestyle.

Aim

The primary objective of this study was to assess the use, user experience and potential health effects of a conversational agent-based eHealth platform (Council of Coaches) implemented in a real-world setting among older adults.

**Methods:**

An observational cohort study was conducted with older adults aged 55 years or older in the Netherlands. Participants were enrolled for 5–9 weeks during which they had access to Council of Coaches. They completed three questionnaires: pre-test, post-test, and at follow-up. After five weeks, an interview was conducted, and participants chose whether they wanted to use the eHealth intervention for another four weeks during the facultative phase.

**Results:**

The study population consisted of 51 older adults (70.6% female) with a mean age of 65.3 years (SD = 7.4). Of these, 94.1% started interacting with Council of Coaches, and most participants interacted once per week. During the facultative phase, 21 participants were still interacting with Council of Coaches. Minimal clinical important differences in quality of life were found among the study population after interacting with Council of Coaches.

**Conclusion:**

Our results demonstrate that eHealth interventions with virtual coaching can be used among older adults. This may increase quality of life for older adults, and decrease their healthcare needs. Future research into such eHealth interventions should take into account the inclusion of sufficient personalised content and the use of a mixed methods study for assessing the eHealth intervention.

## Highlights

• Older adults' use of and user experience with a virtual coaching system are examined.
• Older adults used the eHealth application mostly once a week.
• The eHealth application was easy to use for older adults.
• Most older adults experienced a minimal clinical important difference in health factors.
• For virtual coaching systems it is necessary to have enough personalised content.

## 1 Introduction

The average human life expectancy has steadily increased in recent decades (Gulland, 2014; Suzman et al., 2015). Among the aging global population there has been a concomitant increase in the prevalence of chronic diseases (Suzman et al., 2015; Van Oostrom et al., 2016), which places additional demands on the health care system (Van Oostrom et al., 2016; Van Oostrom et al., 2014). Adopting a healthy lifestyle can reduce a person's risk of chronic disease, and their healthcare burden (Visser, 2000; World Health Organization [WHO], 2005). eHealth interventions are one way to help people adopt a healthy lifestyle (Chatterjee et al., 2019; Tse et al., 2008).

Many emerging eHealth interventions have a focus on behaviour change, for example relating to physical activity, addiction, and weight loss (Dallery et al., 2015). Several review studies found that eHealth interventions are effective in achieving behaviour change towards a healthy lifestyle. For example, one found that eHealth interventions targeting behaviour change in young adults can be effective in the short term (Oosterveen et al., 2017). Another found that in the short term, eHealth interventions are effective at promoting physical activity in older adults (Muellmann et al., 2018).

Embodied conversational agents (ECAs) are computer-generated animated characters that facilitate one-on-one personal interactions with users. ECAs can be included in eHealth interventions to increase user engagement and achieve better outcomes. ECAs are (most of the times) not included to have a more fun eHealth intervention, but to create conversations with the users about helping/supporting them. Scholten et al. (2017) found in their review that including an ECA in an eHealth intervention improved user motivation and duration of participation. A randomized controlled trial (RCT) of an ECA eHealth intervention for healthy living among older adults (*N* = 263) found that the eHealth intervention was more effective at increasing physical activity in the short term than the use of a pedometer. In the long term, this effect was not visible in this study (Bickmore et al., 2013).

Kantharaju et al. (2018) performed a fundamental study within the context of this research, investigating the effect of employing multiple virtual agents to persuade the user who is interacting with the system. The benefit of using multiple virtual agents is that they can discuss a health topic and its benefits with each other, and persuade each other (and any potential bystanders), rather than trying to convince the user directly. Translating this concept to the field of eHealth, in order to convince a user of the importance of a health topic, it may be more effective for two virtual coaches to discuss a health topic, compared to having an individual virtual coach directly persuade the user. This positive effect of vicarious persuasion is one of the core elements of Council of Coaches (COUCH), a new virtual coaching concept that is the topic of evaluation in this article (Op den Akker et al., 2018).

The use of VCSs in older adults has not yet been studied, however this population is at higher risk of chronic disease and in need of effective eHealth interventions. Many VCS studies focus on short-term studies, such as single interaction in a lab setting conducted with a focus group to assess the usability and acceptance of the VCS. However, to better assess the acceptance and user experience, especially in the older adult population, a long-term study of several weeks is needed. A long-term study allows older adults to use the intervention in their homes for a longer period and become comfortable using the technology. Therefore, in this study we focus on the long-term use of a VCS. To apply the renewed framework of evaluating eHealth (Jansen-Kosterink et al., 2016), we address the following objective in our study: to assess the use, user experience and potential health effects of a conversational agent-based eHealth platform in a real-world setting among older adults.

## 2 Material and methods

The detailed methods of this study have previously been published (Hurmuz et al., 2020). Participants were included in this observational cohort study for 5–9 weeks. This study was conducted according to the principles of the Declaration of Helsinki (64th WMA General Assembly, Fortaleza, Brazil, October 2013) and in accordance with the Medical Research Involving Human Subjects Act (Dutch law: Wet medisch-wetenschappelijk onderzoek met mensen). According to this law, this study did not require formal medical ethical approval. This was confirmed by the Medical Research Ethics Committee CMO Arnhem-Nijmegen (file number: 2019-5555).

### 2.1 eHealth intervention

**Fig. 1:**
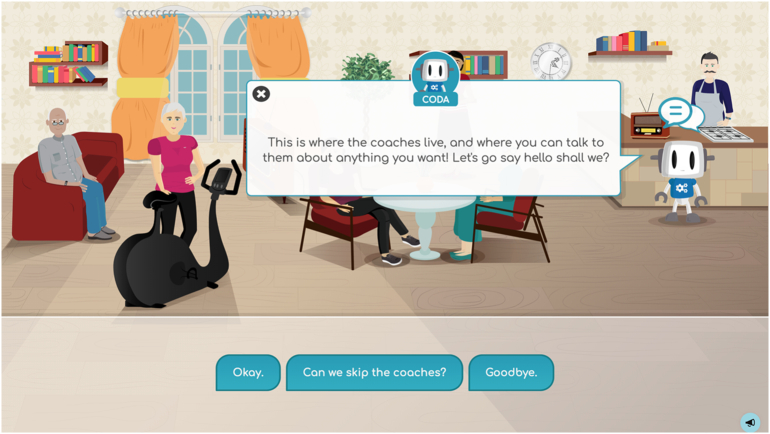
Screenshot of the Council of Coaches' living room. f.l.t.r. Carlos (peer), Olivia (physical activity), Emma (social), Katarzyna (Diabetes), Helen (Cognitive), Coda (helpdesk robot), and François (nutrition) (https://www.council-of-coaches.eu/).

Within the COUCH project (European Union's Horizon 2020 research and innovation program under grant agreement No. 769553), a functional demonstrator (Technology Readiness Level 6) of COUCH (Fig. 1) was developed for older adults, adults with diabetes mellitus type 2 and adults with chronic pain. This eHealth application was developed in the Netherlands. It allows users to have natural language dialogues with a group of virtual coaches. There are a total of 160 dialogues among all coaches. These virtual coaches have their own expertise in several domains: physical activity, nutrition, social, cognition, peer/support, chronic pain and diabetes. When a user is not diagnosed with diabetes mellitus type 2 or chronic pain, the coaches of these domains are not available. The interaction between users and the coaches happens via a text-based user interface; a speech bubble pops up, and the user has several answer options to choose. More information about the dialogue content and the implementation of the dialogues is described in a paper by Beinema et al. (2021).

### 2.2 Study procedure and participants

This study was conducted from January 31 to August 9, 2020, in two rounds. Each round started with an intake and consisted of three phases. The preparation phase (week 1) was the baseline week, with use of an activity tracker but no eHealth intervention. The implementation phase (weeks 2–5) involved the activity tracker and the eHealth intervention. The facultative follow-up phase (weeks 6–9) included the activity tracker and the eHealth intervention, if the user elected to continue.

During intake, participants were informed about the study, received a guideline about COUCH, and were informed that they could interact with COUCH however and whenever they wanted. After the implementation phase, participants were interviewed and were asked whether they were willing to finish the facultative follow-up phase. Beforehand participants were informed that they would receive a small gift to thank them for participating, independent from how actively they participated.

The study population was recruited through advertisements in local newspapers, advertisements on social media, and through snowball sampling. Participants were eligible for this study when they were 55 years of age or older, were able to read and speak Dutch or English, had Wi-Fi connection at home, were willing and able to give informed consent, and were able to clearly see a smartphone or tablet screen.

### 2.3 Outcomes

The primary outcomes of this study were the use of COUCH, user experience with COUCH, and potential health effects. Use was defined as the frequency, duration and interaction (i.e. number of dialogue steps) of use overall, per week, and per session. User experience was measured with questionnaires and semi-structured interviews, both conducted after the implementation phase (T1). These questionnaires consisted of the Technology Acceptance Model (TAM) (Davis, 1989; Davis et al., 1989), the System Usability Scale (SUS) (Brooke, 1996), and the willingness to pay. Finally, the health-related factors were measured with activity tracker data, and with three questionnaires completed at three timepoints: baseline (T0), after the implementation phase (T1) and after the facultative follow-up phase (T2). The three questionnaires were: the EQ-5D-5L (Van Reenen and Janssen, 2015), the six domains of Positive Health (Huber et al., 2016; van Velsen et al., 2019), and the short version of the Self-Management Ability Scale (SMAS-s) (Steverink, 2009). Table 1 gives an overview of all questionnaires used, and an elaborate explanation about the different questionnaires is written down in the protocol (Hurmuz et al., 2020).

**Table 1:** Questionnaires used in study and the timepoint when they were used.

Outcomes	Questionnaires used	T0	T1	T2
User experience	TAM		X	
	SUS		X	
	Willingness-to-pay		X	
Health-related factors	EQ-5D-5L	X	X	X
	Six dimensions of positive Health	X	X	X
	SMAS-s	X	X	X

### 2.4 Data analyses

Quantitative data were analysed with SPSS v.19 Windows (IBM Corp., Armonk, NY). The significance levels were set at 5%. Descriptive statistics, such as frequency, mean, standard deviation and percentages, were used to describe demographics, use and quantitative user experience data (TAM, SUS, willingness-to-pay). Before analysing the log data, some rules were specified:

1. Session duration was defined as number of minutes that the user interacted with coaches without interruption. So, the duration of browsing through the recipe book, or only listening to the radio, were not included in the session duration.
2. Session duration <1 min was not included.
3. Break time within a session was defined as time ≥ 1 min between two interactions. Break times longer than the median were omitted from the duration of the corresponding session.
4. If break time was ≥20 min, the subsequent interaction was considered a new session.

Qualitative user experience data were analysed with ATLAS.ti, v.8 Windows (Berlin, Germany). Interviews were recorded, transcribed, and independently coded by two authors (MH, KF), and discrepancies were discussed until consensus was reached.

Outcomes of the health questionnaires (EQ-5D-5L, the Positive Health tool, and the SMAS-s) were assessed on group and individual levels. On the group level, we assessed normality with histograms. For variables that were normally distributed, we used a linear mixed model analysis with Sidak adjustments. For variables that were not normally distributed, we used the Friedman test. On the individual level, we assessed whether there were minimal clinical important differences (MCIDs) between T0 and T1, T0 and T2, and T1 and T2. In literature we did not find cut-off points for an increase to be clinically relevant for all the health variables we measured. The MCID threshold for EQ-5D-5L was set at 0.074, in accordance with the literature (Jayadevappa et al., 2017). The MCID threshold for the other health variables was set at 25% increase. In literature, we found multiple studies stating that the minimal increase of the baseline value is around 25% to consider it as an MCID (Henderson et al., 2019; Hernandez-Sanchez et al., 2014; Van Hooff et al., 2010).

For activity tracker data, step data below 100 steps per day were removed (this was considered to be non-wear). For each participant, a mean number of steps per day per week was calculated. We divided participants into two groups according to their activity during the baseline week: group A had mean steps per day higher than the median for all participants that week, and group B was lower than the median. Step data was tested for normality with histograms. In group A, there was one week in which the step data was not normally distributed. For this group, so we analysed the data with Friedman test. For the total study population and group B, data was analysed with linear mixed model analysis.

For analysing questionnaires, interviews, and activity tracker data, per protocol analysis was used. If a participant did not interact with COUCH at least once in the implementation phase, their data was omitted. The reason we chose this analysis, was because if someone did not use the eHealth application, (s)he could not give proper answers on the questionnaires and interview questions.

## 3 Results

### 3.1 Participants

of the study began with 51 participants. The mean age was 65.3 years old (SD = 7.4 years), and the majority was female (*N* = 36, 70.6%) (Table 2). After completing the baseline (T0) questionnaire, three participants did not use COUCH, and were not included in the final analyses.

**Table 2:** Demographics of study population (*N* = 51).

		M (SD) or N (%)
Age (M (SD))		65.3 (7.4)
Sex (N (%))		
	Male	15 (29.4%)
	Female	36 (70.6%)
Level of education (N (%))		
	Preparatory secondary vocational education	9 (17.6%)
	Higher general secondary education, pre-university education	16 (31.4%)
	Higher vocational education, university	26 (51.0%)
Living situation (N (%))		
	Married/living together	37 (72.5%)
	Alone	14 (27.5%)
Employment status (N (%))		
	Employed	14 (27.5%)
	Volunteer/caregiver	7 (13.7%)
	Retired	23 (45.1%)
	Other	7 (13.7%)
Health literacy (M (SD))^a^		4.0 (0.6)
Self-reported level of physical activity (N (%))		
	Not at all	1 (2.0%)
	Not at all, but thinking about beginning	2 (3.9%)
	< 2.5 h a week	16 (31.4%)
	> 2.5 h a week in the last six months	9 (17.6%)
	> 2.5 h a week for more than six months	23 (45.1%)
Attitude towards technology (M (SD))^b^		4.5 (1.5)
Type of motivation to live healthy (M (SD))^b^		
	Intrinsic motivation	5.1 (1.0)
	External regulation	2.8 (1.2)
	A-motivation	2.1 (1.3)

a Measured on a scale from 1 (low) to 5 (high).

b Measured on a scale from 1 (low) to 7 (high).

### 3.2 Use of Council of Coaches

During the implementation phase, 48 participants interacted with COUCH at least once. During these four weeks, participants interacted with COUCH on an average of 5.3 days (SD = 3.7). COUCH was used most often during the first week of the implementation phase (week 2, M = 3.3 sessions, SD = 2.2) (see Table 3). During the facultative phase, 21 participants interacted with COUCH on an average of 3 days (SD = 3.0). Reasons given for interacting with COUCH during the facultative phase were: to see whether the coaches had new content (*N* = 10), to receive healthy living advice (*N* = 2), out of curiosity (N = 2), because it was fun (N = 1), and because of promises made to the researcher (N = 1). Reasons for not interacting during this phase were personal (no time, sickness, already very active, no motivation) (*N* = 10), not receiving added value from interaction (*N* = 6), content-related (too little, too general) (*N* = 5), or technology-related (too difficult) (N = 5). One participant indicated not interacting with COUCH because of having real-life coaches, and one because of the outbreak of the COVID-19 pandemic. In the third week of the facultative phase (week 8), COUCH was used most often (M = 2.0 sessions, SD = 1.4) (see Table 3). For every week, except week 2, most participants interacted once with COUCH.

**Table 3:** COUCH use data.

Week	N	Mean (SD) number of sessions	Range min-max number of sessions	Mean (SD) duration in minutes per session	Range min-max duration in minutes per session	Mean (SD) number of interactions per session	Range min-max number of interactions per session
1	–	–	–	–	–	–	–
2	44	3.3 (2.2)	1–11	7.2 (5.1)	1.0–23.1	114.1 (82.7)	9–471
3	33	2.0 (1.4)	1–6	7.4 (5.6)	1.1–23.1	114.0 (92.4)	6–448
4	25	1.5 (0.8)	1–4	7.9 (6.4)	1.1–28.8	122.4 (76.5)	19–339
5	22	2.0 (1.5)	1–7	5.8 (5.9)	1.1–26.7	90.7 (75.8)	18–388
6	17	1.8 (1.1)	1–5	5.2 (4.1)	1.2–15.4	91.6 (66.8)	16–282
7	10	1.6 (0.8)	1–3	5.0 (2.9)	1.8–11.2	79.8 (36.4)	36–173
8	7	2.0 (1.4)	1–4	4.9 (2.4)	1.4–8.4	81.0 (51.2)	13–232
9	7	1.7 (1.1)	1–4	5.0 (4.4)	1.2–13.9	80.3 (70.3)	21–227

During interviews, participants were asked for reasons to interact with COUCH and to not interact with COUCH. Regarding reasons to interact with COUCH, the most common response was to become more physically active (*N* = 13). Other reasons related to healthy living (*N* = 19) included: to lose weight, and to receive advice related to healthy eating and health conditions. As one participant said: *“In some areas you do notice that you are getting older and that your body abandons you in those areas.”* (P-1). Other reasons were related to participants' daily routine (*N* = 5), such as getting knowledge about and being aware of their health; social participation (*N* = 3), such as expanding social contacts, mental health (N = 3), such as being informed about mental wellbeing; and quality of life (*N* = 2), such as feeling well-balanced. Other reasons (*N* = 9) included: just for fun, helping researchers, and curious about the technology. Six participants did not indicate any reasons to interact with COUCH.

Reasons to not interact with COUCH were mostly related to the technology (*N* = 45), such as the content of the coaches and difficulty logging in. In total, eight participants indicated they had no reasons for not interacting with COUCH: *“I do not have a reason. I think health is an important subject, and knowledge about this is very important.”* (P-25). Other reasons were not having enough time to interact with COUCH (*N* = 3), starting a new intervention (*N* = 1), or related to participants' bodily functioning (*N* = 5), social participation (*N* = 2), and quality of life (N = 1).

### 3.3 User experience

The usability of COUCH was scored with a mean of 51.4 (SD = 20.0, *N* = 46). This means that the usability of the system was marginally acceptable according to the participants. During interviews, 15 participants indicated they experienced some problems with COUCH: too slow or technical issues. Most participants found it easy to use COUCH (*N* = 24 vs. *N* = 9 difficult), and two indicated that it was difficult in the beginning, but after a while it was easy to use: *“In the beginning, I have to be honest with you, my daughter helped me. I am not very into this. She said you have to do it this and this way, and then you master it.”* (P-28).

**Fig. 2:**
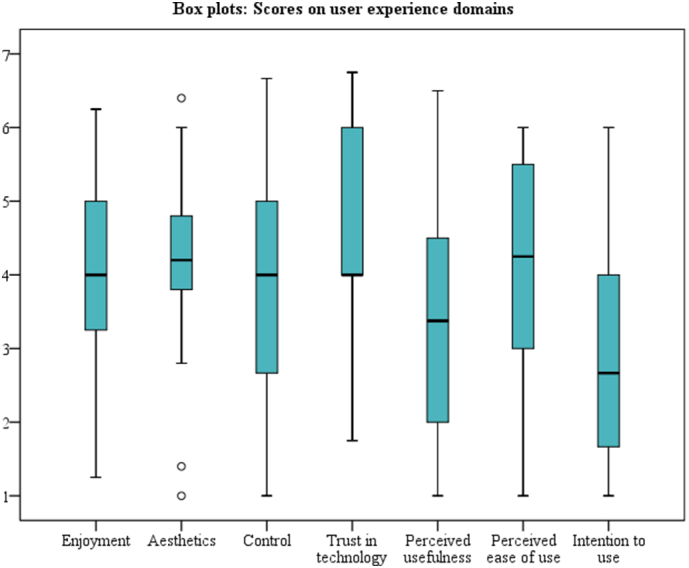
Box plot representation of user experience on seven domains of the TAM (N = 46).

Regarding the user experience measured with the TAM, participants were mostly neutral about COUCH. Participants were most positive about the trust in COUCH (M = 4.6, SD = 1.0, scale 1–7), and least positive about the intention to use COUCH (M = 2.9, SD = 1.7, scale 1–7). Table 4 shows the mean of each user experience domain, and the percentages of participants that were positive, neutral and negative towards each domain. Fig. 2 shows the box plot of each domain.

**Table 4:** User experience assessed on seven domains of the TAM (*N* = 46).

User experience domains	M (SD)	% negative	% neutral	% positive
Enjoyment	3.8 (1.2)	17.4%	80.4%	2.2%
Aesthetics	4.3 (1.1)	4.3%	82.6%	13.0%
Control	3.9 (1.6)	21.7%	63.0%	15.2%
Trust in technology	4.6 (1.0)	2.2%	69.6%	28.3%
Perceived usefulness	3.4 (1.6)	34.8%	56.5%	8.7%
Perceived ease of use	4.1 (1.5)	17.4%	67.4%	15.2%
Intention to use	2.9 (1.7)	47.8%	43.5%	8.7%

Note: These user experience domains are measured on a scale from 1 (negative) to 7 (positive).

Nine participants (19.6%) indicated that they are willing to pay for using COUCH. The average price a participant was willing to pay was €6.15 per month (N = 13). Twelve participants indicated they would recommend COUCH to others, mostly for people who, for example, have little knowledge about healthy living, are lonely, or people who have difficulties changing or want to change their lifestyle (*N* = 10): *“If someone wants to change his lifestyle, I would recommend this. There are good tips about food and drinks, there are recipes etc., and I heard good tips to prevent dementia.”* (P-47). Other examples for why to recommend COUCH were because it is helpful (*N* = 5), it gives the user discipline to follow advice (*N* = 3), and because with COUCH the user has easy access to health advices (*N* = 2).

However, 30 participants would not recommend COUCH to others, and 5 participants were neutral towards this. The most common reason (*N* = 15) for not recommending COUCH was that the coaches give overly generalized information: *“The questions are being asked from situations not corresponding to mine.”* (P-20). Other reasons mentioned more than three times were about: limited content (*N* = 9), childish/patronizing conversations (*N* = 6), and difficulty logging in (*N* = 4).

Regarding COUCH's user experience, 19 participants had a good experience: *“That* [interaction with system] *was absolutely great.”* (P-15), *“The ease of use is fine.”* (P-46), *“That works well, the interaction with the coach, it is funny, it is nicely built.”* (P-47). Six participants liked the appearance of the system: *“I really like the way it looks; I really like how the system is built.”* (P-1). Participants liked the recipes (*N* = 4) and advice given by the coaches (*N* = 3): *“I came across a nice recipe book. It was very concrete, and I could retrieve really nice dishes from this book.”* (P-10), *“The tips she* [Emma, the social coach] *gives are good for being socially active. You get confronted with your own situation.”* (P-47). Two participants said interacting with the coaches was fun. Others responded: friendly coaches, clear conversations, good interaction between the coaches, especially François (nutrition coach) was lovely to interact with, the way the coaches talk to the user is good, the user has the control, fun radio, nice brain quizzes, and good intent of the application, (*N* = 1 each).

However, 24 participants also had some critical points, mostly regarding the content: too limited content and general advices (*N* = 9): *“It is good to exercise, yeah I know that too. But help me with my own situation. I tried, but the dialogues are limited.”* (P-45), the social talks were not liked a lot (*N* = 6): *“Just the social talks, I am not very into that. … It is not needed for me.”* (P-26), too much repetition in the dialogues (*N* = 1), no need for knowing François' food preferences (N = 1), childish explanations (N = 1), not interesting in general (N = 1), no concrete tips/advices (N = 1), too many step-by-step explanations (N = 1), unilateral stories (N = 1), sometimes wrong answer options (N = 1), and hard to interact with the coaches because of pre-programmed dialogues (N = 1). Seven participants found it cumbersome that they had to log-in each time they wanted to interact with the coaches. Furthermore, some responded that it was too robotic and it did not stimulate the user (*N* = 3 each). The following comments were mentioned twice: did not like the lay-out, no possibility to ask questions, too simple, childish in general, the coaches ask for information that is too personal, not liking the interaction with the coaches, and annoying that after each log-in the user does not continue where user was left. Finally, some participants had other comments: did not like the radio, no personal connection with François, crappy system, did not like Emma, Emma assumes it is a problem when you have little social contacts, paternalistic coaches, and not interactive.

### 3.4 Potential health effects

For measuring the potential health effects, three questionnaires were used. Table 5 shows the mean scores of all health variables at T0, T1 and T2. Two variables were not normally distributed: perceived health state measured with the EQ-5D-5L and the Positive Health domain mental health. For these variables, the Friedman test did not show any significant effects between the different measurement points. For all other variables, the mixed model analyses showed that in two SMAS-s domains there was a significant difference: the investment behaviour domain (*P* = 0.013, F = 4.588, df = 88.184) and the self-efficacy domain (*P* = 0.028, F = 3.737, df = 88.246). For both variables, the best model fit (measured with the Akaike's Information Criterion) was with the covariance structure Compound Symmetry. The Sidak multiple tests adjustment showed that for the investment behaviour domain, there was a significant effect between T0 and T2. The mean increase was 5.542 (SE = 1.912, *P* = 0.014). For self-efficacy there was a significant increase between T1 and T2, with a mean increase of 4.581 (SE = 1.725, P = 0.028).

**Table 5:** Mean (SD) of health variables at T0, T1, and T2.

Health variables		M (SD) at T0 (*N* = 48)	M (SD) at T1 (*N* = 47)	M (SD) at T2 (*N* = 42)
Perceived health state		0.83 (0.15)	0.84 (0.15)	0.86 (0.15)
Perceived health state on a VAS		78.1 (15.4)	79.1 (14.3)	81.4 (13.0)^b^
Positive Health domains				
	Bodily functions	7.0 (1.6)	7.3 (1.5)	7.5 (1.5)^b^
	Mental health	7.5 (1.4)	7.7 (1.4)	7.9 (1.3)^b^
	Meaning	7.7 (1.6)	7.9 (1.6)	8.1 (1.3)^b^
	Quality of life	7.7 (1.5)	7.9 (1.5)	8.0 (1.3)^b^
	Social participation	7.7 (1.4)	7.9 (1.7)	8.1 (1.3)^b^
	Daily routine	8.1 (1.3)	8.3 (1.4)^a^	8.4 (1.3)^b^
SMAS-s domains				
	Taking initiatives	65.1 (14.6)	67.9 (13.0)	70.5 (16.3)
	Investment behaviour	69.4 (14.5)^c^	70.8 (14.1)	74.9 (14.3)^c^
	Variety	61.5 (19.2)	60.7 (16.3)	64.1 (16.3)
	Multifunctionality	63.2 (14.2)	61.8 (15.4)	62.1 (14.6)
	Self-efficacy	70.3 (14.4)	67.4 (15.4)^c^	71.6 (15.5)^c^
	Positive frame of mind	58.5 (17.0)	62.3 (18.1)	60.6 (15.4)
SMAS-s total score		64.7 (11.3)	65.2 (11.3)	67.3 (11.1)

a N = 46.

b *N* = 41.

c Significant (*p* < 0.05).

On the individual level, 41 of 47 participants (87.2%) experienced an MCID in at least one health variable during the whole study period. From T0 to T1, most MCIDs were found in the SMAS-s domain positive frame of mind (*N* = 11), followed by the Positive Health domains bodily functions (*N* = 10) and meaning (*N* = 8). Looking at the health scores at T0 and T2, most MCIDs were found in both perceived health state measured with the EQ-5D-5L and the SMAS-s domain taking initiatives (*N* = 11), followed by the SMAS-s domain variety (*N* = 10). From T1 to T2, most MCIDs were found in the SMAS-s domain self-efficacy (*N* = 9), followed by the SMAS-s domains investment behaviour and positive frame of mind, and the perceived health state (*N* = 8).

**Fig. 3:**
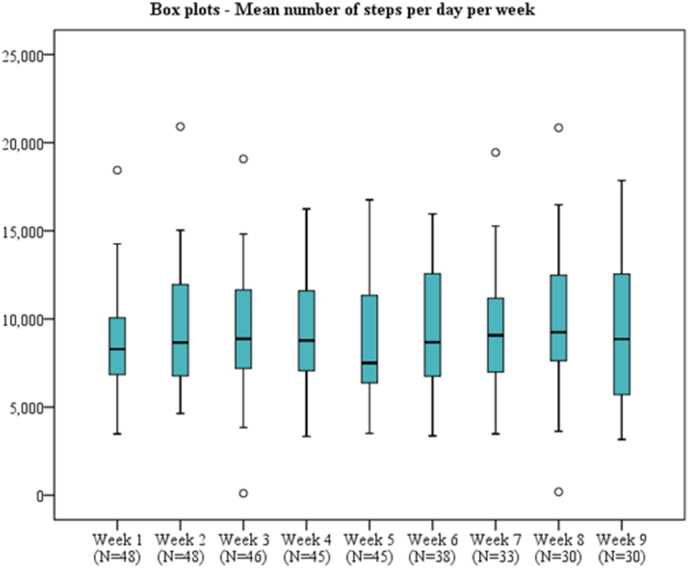
Activity tracker data: Box plot representation of mean steps per day per week the in total study population.

During the baseline week, mean steps per day ranged between 3475 and 18,440 steps, and median steps this week was 8290 steps (IQR = 6793 - 10,215). Fig. 3 shows the box plots of mean steps per day for each week. Mean steps per week increased each week for the total study population. For group B (participants with mean steps below median during baseline week) this increase was maintained. For group A (above median), in four weeks there is an increase compared to the baseline week. These increases were not significant with linear mixed model analyses and Friedman test. However, during the interviews, some participants mentioned things about being more physically active (*N* = 3). For example: *“I think I exercised more, not only because of wearing the Fitbit activity tracker, but also as a result of talking to Olivia, I, yeah, wanted to accomplish my goals, unconsciously.”* (P-4), *“I now live temporarily in an apartment, and I always took the elevator, but now I always take the stairs.”* (P-8). Taking the stairs instead of the elevator was one of the physical activity coach's daily tips.

Regarding healthy living in general one participant said: *“If he* [François, the nutrition coach] *gave tips, they were good tips. Things I already know, like do not eat too much salt and those kind of things. So, for me no added value. But as a system, I think it is also for people that need to start with the basics. And that is really good.”* (P-26). Two other participants indicated that they are more aware of the importance of being socially active as a result of using COUCH: *“Yes, that's important for sure. To develop yourself, otherwise if you are lonely, the loneliness becomes even more. … Because of COUCH I understand the importance of this.”* (P-47).

## 4 Discussion

The aim of this study was to assess the use, user experience, and potential health effects of a conversational agent-based eHealth platform implemented in a real-world setting among older adults. We found three main findings related to our study objective. First, regarding the use of COUCH, in almost all weeks, most older adults that interacted with COUCH did so once per week, and the number of participants declined over time. This confirms the law of attrition in eHealth studies, as described by Eysenbach (2005). The law of attrition has two aspects: the first is about loss to follow-up (i.e. not using the eHealth intervention and not completing questionnaires/interviews), and the second is about non-use (i.e. not using the eHealth intervention, but completing questionnaires/interviews) (Eysenbach, 2005). In our study, both types of attrition were present. A possible explanation for the attrition here can be due to the content of the eHealth application. Because of the mixed methods used in the study, we gathered more qualitative information about the eHealth application, which revealed that the content of the different coaches was a topic of frequent discussion. Most participants thought it was not personalised to their own situation or there was not enough content that warranted continued use of the application. Thus, it is likely that the available content of the coaches influenced the use of the system. This suggestion is supported by two other papers that have analysed the results of this same study from different perspectives. The work of Ter Stal et al. (2021) described which of the coaches in the COUCH system was preferred most/least often and why. This work shows that most older adults did not have a preference for one specific coach, but the coach that is mentioned most often was Olivia (the physical activity coach). The main reason given for this was the content available for Olivia. Her content was perceived positively, because of the feedback she gave, the realistic goals she gave, and the concrete tips she gave, and in fact Olivia was the coach that has the most content in terms of defined dialogue steps. The coach who was mentioned most often as the least preferred one, was Carlos who is not a real coach but a peer. A lack of content and an absence of his personality were cited as reasons for Carlos being least preferred. This paper also shows that the participants were more positive about the different coaches at first sight compared to after four weeks of using the COUCH system (Ter Stal et al., 2021). The second paper, from Beinema et al. (2021) looks specifically at the difference in lengths of interaction in situations where the user chooses the topic of discussion versus situations in which the system suggests a topic. This paper provides a more in-depth analysis of the dialogue types which includes the participants from our study and the study conducted in Scotland. It shows among other things that the acceptance rate of coaching dialogues (e.g. dialogues focusing on tips, feedback, goal-setting) was higher than the acceptance rate of social dialogues (e.g. dialogues focusing on small talk, coaches' background stories) (Beinema et al., 2021). All this demonstrates that for COUCH and other eHealth coaching interventions to be implemented in the real-world situation, the coaches need to focus more on the user's situation to provide personalised content. For example, by gathering information about the user's living situation, hobbies, physical activities, or diet pattern, virtual coaches can give more targeted advice that is relevant to the user.

The COUCH system was easy to use for older adults, and older adults were mostly positive about trust in the technology. An important prerequisite for eHealth technologies to be used by the target group, is that they must be easy to use (Davis, 1989; Huygens et al., 2016; O'Connor et al., 2016).With only the quantitative data (TAM in this case), we could not state that COUCH was easy to use for the participants, as most of them scored perceived ease of use neutral in the questionnaire. However, we asked the participants during interviews whether the use of COUCH was easy, and whether they experienced any problems with its use. These interviews showed us that a total of 72.7% of 33 participants found it easy to use COUCH. This agrees with the published literature, which demonstrates ease of use of VCSs among older adults (Albaina et al., 2009; Jegundo et al., 2020; Mostajeran et al., 2019; Ofli et al., 2016). Our study also demonstrates that older adults have trust in the VCS, which indicates that VCSs may be a solution to achieve behaviour change in older adults, as long as it is easy to use and there is enough personalised content.

Finally, on the group level, no major potential health effects were found after interacting with COUCH. However, looking at the individual level, MCIDs were achieved in health variables. On the group level, participants improved their self-management abilities in investment behaviour and in self-efficacy. This means that after interacting with a VCS, older adults may be better at investing in resources to benefit in the long-term, and better at being conscious about managing these resources to achieve a healthier life (Schuurmans et al., 2005). However, these are potential health effects, as there was no control group in our study. When we look at the average health state of our population, it indicates a quite high quality of life. During interviews, some participants mentioned that they are already very active and living healthy. We could also see this in participants' self-reported physical activity measured in the baseline questionnaire, and the median number of steps during the baseline week. Almost half of the population indicated they are active for more than 2.5 h per week for more than six months. Only three participants indicated not being active at all. This high baseline health status could have influenced our results of the small potential health effects found on group level. However, even though the average health scores at baseline were quite high, almost all participants experienced an improvement in one or more health variable. For future research on potential health effects of an eHealth intervention, we recommend to assess the MCIDs on an individual level. Little was found in the literature towards the health effect of VCSs on older adults. A recent review of VCS for older adults found that some RCTs showed VCS to be effective, and some showed no significant effects (Bevilacqua et al., 2020). For future research, more studies are needed to assess the effectiveness of VCSs, with a focus on MCIDs to assess the system on an individual level. However, it is also necessary to establish a standardized method to measure MCIDs in health variables, in order to assess the clinical relevance of health interventions.

### 4.1 Strengths and limitations

An important strength of our study is the mixed use of methods including quantitative and qualitative measurements. Looking only at the quantitative data, an incorrect conclusion could be derived about COUCH. For example, looking at the SUS, COUCH scored on average low. However, previous research has shown that it is not appropriate to only use the SUS for assessing eHealth usability (Broekhuis et al., 2019). With the data gathered through interviews, we better understood the data measured with questionnaires. When we asked about the usability of and interaction with COUCH during the interviews, most older adults did not experience problems with this. In this stage of the eHealth application, where iterative lab tests are already performed and the product is ready for testing in a real-world setting (Jansen-Kosterink et al., 2016), a mixed methods study has added value compared to only conducting quantitative or only qualitative measurements. For future research with eHealth applications in the same stage, we recommend to use these mixed methods. Furthermore, we noticed that participants were more or less neutral when completing the questionnaires, but when asking about this in interviews, they said that this was because they had other expectations towards COUCH. Some thought they would be coached by a human coach behind the computer, or that COUCH would directly help them to lose weight. For future research, to avoid this mismatch between expectations and reality, we recommend to give attention to expectation management beforehand. In our study we tried to give clear information to the participants about the study and the eHealth intervention itself, however, it appears that more information is needed about what users can expect from the eHealth intervention.

This study had some limitations. As expected (Hurmuz et al., 2020), selection bias was an issue. Possible participants were informed about this study with advertisements. When they were interested in participating, they contacted the first author. This might be the reason for having a study population with older adults who are mainly intrinsically motivated towards healthy living, and have on average a high health literacy. Bickmore et al. (2010) found that patients with low health literacy are more positive towards accepting ECAs compared to patients with high health literacy. As our study population had a high health literacy at baseline, it might have influenced our participants' opinions towards accepting COUCH. Another limitation is that participants felt there was not enough personalised content, which affected their opinion about COUCH. At this moment, COUCH is not a medical device. When the coaches will have more personalised content, this should be reviewed again. Furthermore, it takes a lot of time to write the coaches' dialogues. Within COUCH, there is a considerable amount of content, but, if participants started by exploring all the dialogues on the first day, there was not a lot of new content coming in for the following weeks. Finally, during this study, the COVID-19 outbreak reached the Netherlands. Due to this, the rest of the study was performed remotely: the equipment participants needed were sent via post, and the intake and the interviews were conducted by phone. Nonetheless, we do not think the outbreak and change in study procedure influenced our results. Since the social coach Emma had advice to meet other people, which was no longer applicable for the situation participants were in, a disclaimer was added in the system about this and these dialogues were changed as soon as possible.

## 5 Conclusions

To conclude, older adults interacted with COUCH once a week, found it easy to use, and experienced MCIDs on the individual level in one or more health variable. Our results show that a VCS can be implemented among older adults to motivate them to adopt a healthy lifestyle. This may decrease the risk of being affected by chronic diseases and the burden on the health care system. Older adults are willing to use such eHealth interventions for improving their health and lifestyle if there is sufficient personalised content. From this study, we can derive two important implications for future research. First, when designing VCSs for older adults, it is important to include personalised content. Second, when studying eHealth systems that are in the same development stage as COUCH, a mixed methods study is more valuable than either quantitative or qualitative methods alone.

## Funding

This study was supported by the Council of Coaches project funded by the 10.13039/501100000780European Union's 10.13039/100010661Horizon 2020 research and innovation programme under grant agreement No. 769553. The funding source of this study had no role in the study design, data collection, data analysis, interpretation of the data, writing of the report, and in the decision to submit the paper for publication.

## Declaration of competing interest

The authors declare that they have no known competing financial interests or personal relationships that could have appeared to influence the work reported in this paper.
